# Supplementary material for: Anti-Hyperuricemic Effect of Anserine Based on the Gut–Kidney Axis: Integrated Analysis of Metagenomics and Metabolomics
Source: Nutrients. 2023 Feb 15;15(4):969. doi: 10.3390/nu15040969 (PMC9964072; doi:10.3390/nu15040969)
Supplement: Supplementary file 1 [file nutrients-15-00969-s001.zip › nutrients-2153955 - supplementary.pdf]

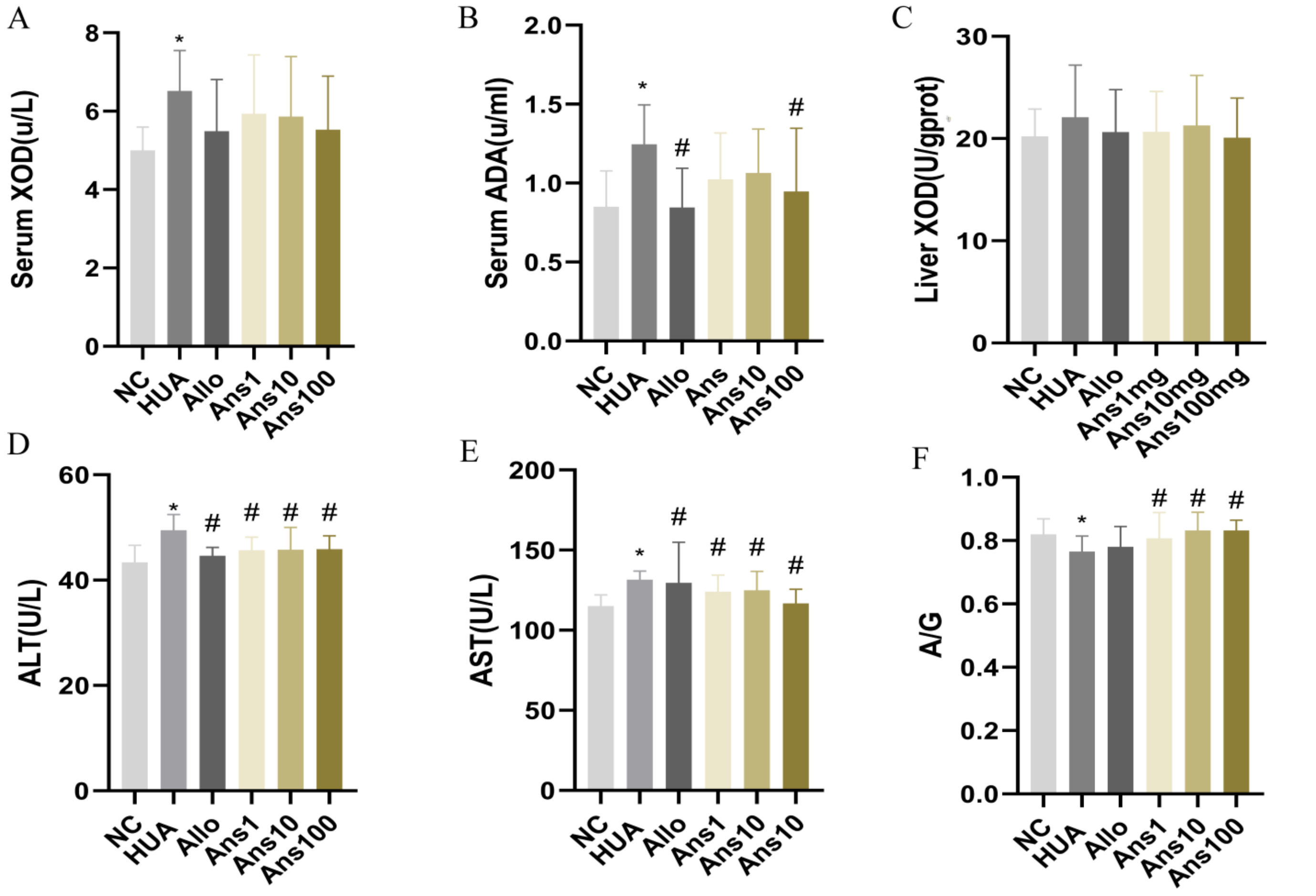

Supplementary Figure S1. Effect of anserine on uric acid production and liver function-related indicators. The level of (A) serum ADA; (B) serum XOD; (C) liver XOD; (D) ALT; (E) AST; and (F) A/G; **P*<0.05 indicates that the difference is significant when compared to the NC group. #*P*<0.05 indicates that the difference is significant when compared to the HUA group

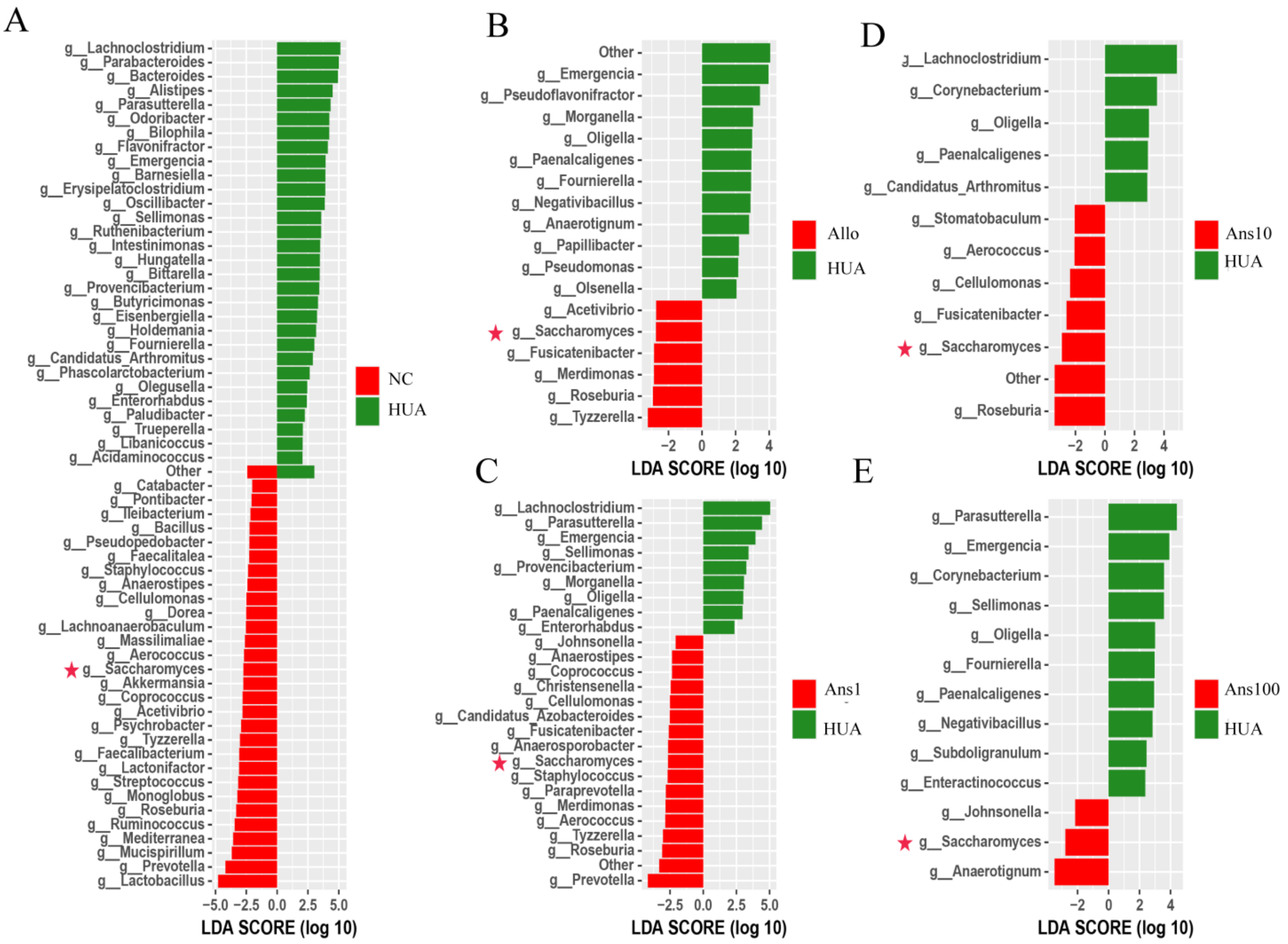

Supplementary Figure S2. Using the Lefser method obtained differential genus (LDA>2) identification between the HUA group and the NC group, Allo group, Ans1mg, Ans10mg and Ans100mg group. These five comparisons yielded a total of 79 different genera. Figure 2A-F refer to HUA vs. NC, HUA vs. Allo, HUA vs. Ans1mg, HUA vs. Ans10mg, HUA vs. Ans100mg. (P<0.05)

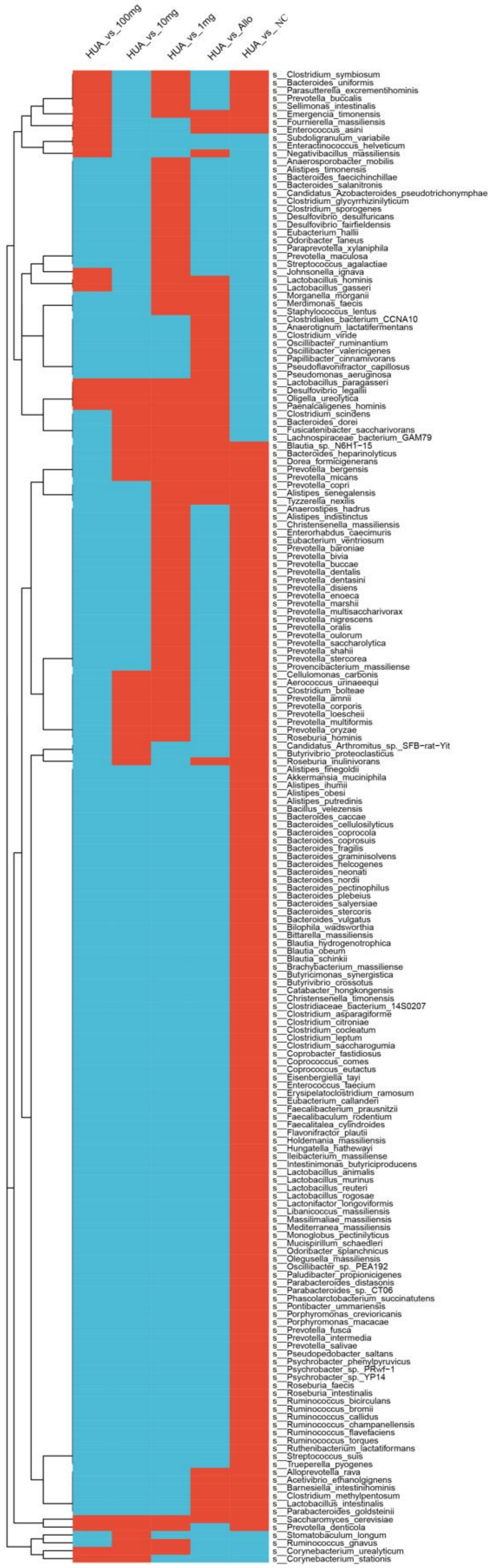

Supplementary Figure S3. Using the Lefser method obtained differential species (LDA>2) identification between the HUA group and the NC group, Allo group, Ans1mg, Ans10mg, and Ans100mg group. These five comparisons yielded a total of 189 different species. (*P*<0.05)

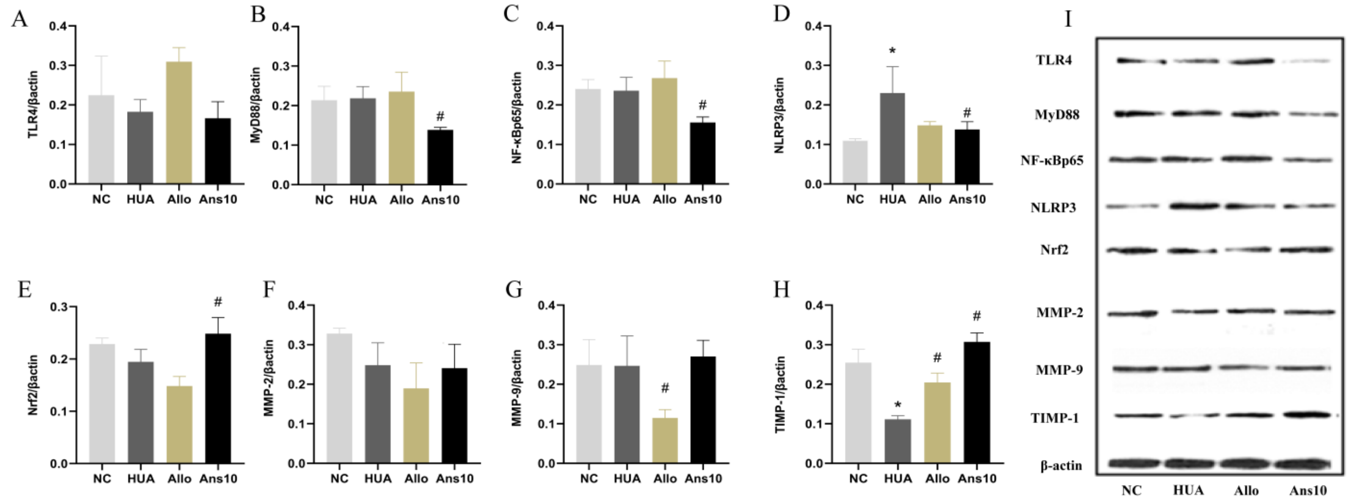

Supplementary Figure S4. Annotation of genes in different groups in the KEGG database. Supplementary Fig 6. A shows the pathways annotations of level 1. The relative abundance of the Metabolism pathway was the highest among the six groups, followed by cellular processes. Supplementary Fig 6. B shows the pathways annotations of level 2. The KEGG level 2 includes 42 subdivided pathways, and the differential analysis obtained 25 differential pathways. Most of the pathways were more abundant in the NC group than in the other five groups, and the most significant differences were in Endocrine and metabolic diseases. In addition, the HUA group had a significantly lower abundance of biosyntheses of other secondary metabolites and the metabolism of terpenoids and polyketides than the NC, Allo, and Ans1/10/100mg groups. (*P*<0.05)

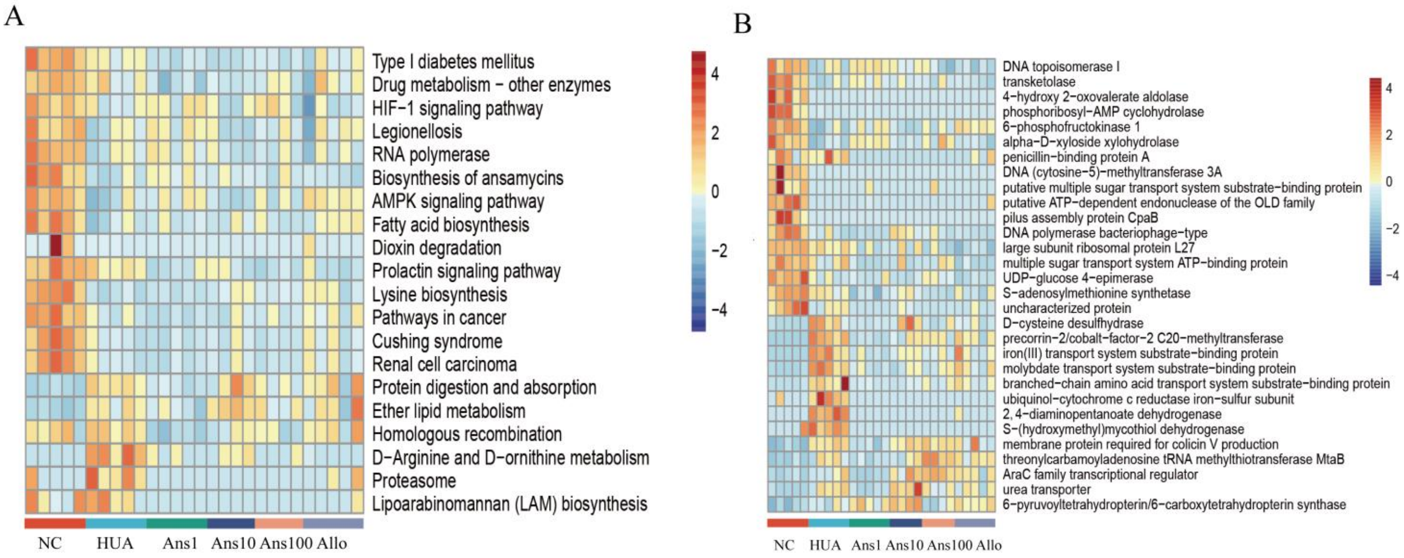

Supplementary Figure S5. The enrichment of genes in different groups in the KEGG map and KO. Supplementary Fig4.A-B show the relative abundance of the top 20 differential maps and the top 30 differential KOs among the six groups. (P<0.05)

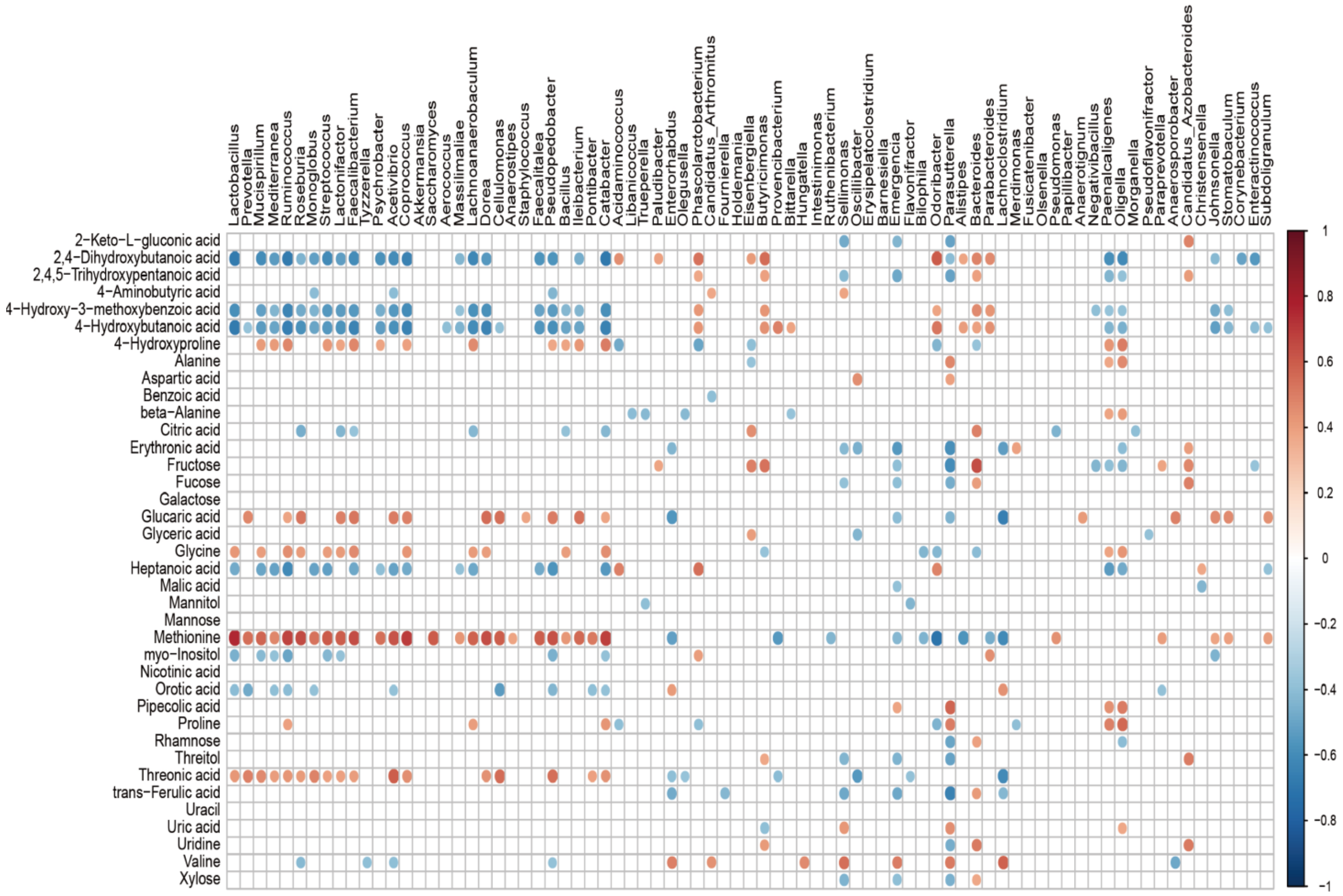

Supplementary Figure S6. Association analysis between metabolites and differential genera.

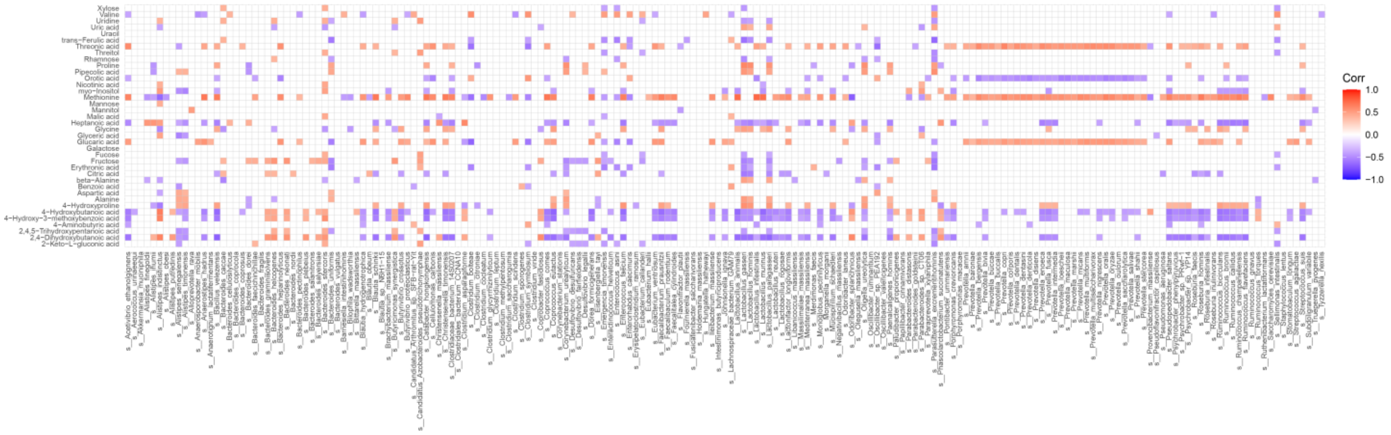

Supplementary Figure S7. Association analysis between metabolites and differential species.
